# The Resurgence of Pertussis in Tuscany (Italy): A Six-Year Retrospective Epidemiological Analysis

**DOI:** 10.3390/pathogens15030326

**Published:** 2026-03-18

**Authors:** Sara Boccalini, Manuela Chiavarini, Alice Dell’Acqua, Beatrice Conti, Zhanna Tumanova, Alessandra Picelli, Vanessa Verniani, Daniele Borchi, Lorenzo Latella, Saverio Checchi, Matteo Bastiani, Barbara Rita Porchia, Daniela Senatore, Giovanna Bianco, Paolo Bonanni, Angela Bechini

**Affiliations:** 1 Department of Health Sciences, University of Florence, 50134 Florence, Italy; 2 Postgraduate School in Hygiene and Preventive Medicine, University of Florence, 50121 Florence, Italy; 3 Regione Toscana, Direzione Sanità, Welfare e Coesione Sociale, 50139 Florence, Italy

**Keywords:** epidemiological surveillance, waning immunity, adolescent immunization, maternal vaccination, public health preparedness, epidemic, acute respiratory infection

## Abstract

Pertussis, caused by *Bordetella pertussis*, remains a public health concern despite long-standing vaccination programs. After a marked decline during the COVID-19 pandemic, a resurgence was observed in Europe and Italy, with a sharp increase in 2024. This study describes pertussis epidemiological trends in the Tuscany Region (Italy) from 2019 to 2024 to identify high-risk groups and inform prevention strategies. A retrospective population-based analysis was conducted using cases reported to the national surveillance system (PREMAL). Incidence rates were calculated using ISTAT population data, and demographic, temporal, and clinical characteristics were analyzed. Overall, 669 cases were reported (mean annual incidence rate: 3.03/100,000 (IC 95% 2.47–3.59; period incidence rate: 18.2/100,000 (IC 95% 16.81–19.56)), with 89% occurring in 2024 (16.34/100,000 (IC 95% 15.03–17.65)). No sex differences were observed, and most cases were reported in Central Tuscany (64%). Children under 15 years accounted for 87% of cases. The highest incidence was observed among 10–14-year-olds, while infants < 1 year, particularly those under 4 months, showed the highest burden in narrower age strata. Hospitalizations occurred in 12.6% of cases, decreasing substantially in 2024. The 2024 resurgence likely reflects waning immunity, disruptions to routine vaccinations during the pandemic, and reduced pathogen circulation in previous years due to containment and isolation measures related to the pandemic. Strengthening surveillance and improving booster and maternal vaccination coverage are essential to protect vulnerable populations.

## 1. Introduction

Years after the implementation of specific vaccination programs, pertussis still represents a significant public health concern at the global, European, and national levels [1,2]. Pertussis, or whooping cough, is an acute respiratory infection caused by *Bordetella pertussis*, a highly contagious and droplet-transmitted pathogen [3]. It can affect all age groups, but the most severe manifestations and complications are observed in newborns and infants [3,4]. The disease typically evolves through three phases: catarrhal, with flu-like symptoms; paroxysmal, characterized by intense and persistent coughing, the typical inspiratory “whoop” and possible episodes of cyanosis and post-tussive vomiting; and finally, a convalescent phase, during which the cough may persist for weeks or months. Complications, especially in newborns, include pneumonia, apnea, seizures, hypoxic encephalopathy, and, in the most severe cases, death [5]. Specifically, immunized or unimmunized infants of less than 6 months of age are the most at risk, followed by people with comorbidities such as asthma, chronic obstructive pulmonary disease or immunosuppression [6]. Moreover, other groups require particular attention in terms of preventive strategies, such as pregnant women, healthcare workers and people working/living with newborns and infants [7].

In Europe, pertussis currently follows a cyclical trend with epidemic peaks every 3–5 years, in addition to seasonal fluctuations, with most cases occurring in spring and summer [2,6]. After a period of relative stability between 2010 and 2015, an increase in cases was observed during 2016–2019, particularly in Northern European countries. During the COVID-19 pandemic (2020–2022), containment measures drastically reduced bacterial circulation, reaching a historic low in 2021. However, in 2023–2024, a new increase was recorded, attributable to reduced vaccination coverage, delays in booster doses, and declining herd immunity [6].

In the past decade, Italy followed a similar pattern. Data from the Istituto Superiore di Sanità (ISS) reveal an increase from approximately 400–500 annual cases between 2010 and 2015, peaking at 670 in 2014, to over 900 during 2016–2018 [8]. The onset of the COVID-19 pandemic was followed by a decline to less than 200 cases in 2020 and even fewer between 2021 and 2023. Nevertheless, 2024 was marked by a resurgence of more than 4600 cases. It is important to note that pertussis incidence is likely underestimated due to diagnostic challenges and the overlap with other respiratory infections, especially in case of milder forms of the disease [9,10].

While national-level data are available, regional analyses remain scarce. The epidemiological pattern observed in Tuscany, a region in Central Italy, is consistent with that described at the national level [11]. Between 2010 and 2018, fewer than 100 cases per year were reported, with a maximum of 115 cases recorded in 2017. The present study provides a descriptive analysis of reported pertussis cases in Tuscany during the 2019–2024 observation period. Specifically, it characterizes the distribution of cases by person, place, and time and describes reported complications and population groups represented among notified cases in order to inform prevention and control activities at the regional level.

## 2. Materials and Methods

This retrospective, population-based study was conducted on pertussis cases reported in the Tuscany Region (Italy) between 1 January 2019 and 31 December 2024. As of 1 January 2024, the resident population of Tuscany was 3,660,530 inhabitants, distributed across three Local Health Authorities (LHAs): Central (population 1,605,995), North-West (population 1,245,397), and South-East (population 809,138) [12]. The Tuscany Center LHA includes the provinces of Florence, Prato, and Pistoia; the Tuscany North-West LHU comprises the provinces of Lucca, Pisa, Massa Carrara, and Livorno; and the Tuscany South-East LHA covers the provinces of Arezzo, Siena, and Grosseto.

Case-specific data were obtained from “Sistema di segnalazione delle Malattie infettive” PREMAL, the national surveillance system for notifiable infectious diseases, which was provided by the Ministry of Health [13]. It relies on routine reporting by healthcare providers and Local Health Authorities (ASLs), without active case finding. Standardized forms collect demographic and clinical information, including, since 2024, the degree of severity of cases. Laboratory confirmation and vaccination status are not consistently available. Incidence estimates are calculated using the total resident population as the denominator, without adjustment for vaccination coverage or underlying susceptibility. The strengths of PREMAL include nationwide coverage, longitudinal data collection, and the ability to monitor trends over time and across age groups. The limitations are inherent to passive surveillance: case ascertainment depends on clinical recognition and reporting practices, which may vary over time and by region; mild or subclinical cases are likely underreported. Consequently, passive surveillance cannot directly capture the true disease burden. Despite these limitations, PREMAL provides a cost-effective and sustainable tool for population-level monitoring and public health decision-making.

Population denominators were obtained from the Italian National Institute of Statistics (ISTAT), which provides annual estimates of the resident population by demographic and geographic characteristics [14].

The study population included all pertussis cases among Tuscany residents reported in PREMAL. Available variables included: year of notification, sex, age group, Local Health Authority (LHA) of residence, and hospitalization status. Cases were classified according to the national case definition of pertussis established by the Ministry of Health, which integrates clinical, laboratory, and epidemiological criteria [15].

Descriptive analyses examined the distribution of cases by year, sex, age group, and LHA of residence. Hospitalizations were analyzed both as absolute numbers and as proportions of reported cases. Incidence and hospitalization rates per 100,000 residents were calculated using annual population denominators provided by ISTAT. Period incidence rates across multiple years of analyses were calculated by using the average resident population over the observation period as the denominator.

For each estimated incidence rate, the corresponding 95% confidence interval (CI) was calculated and reported to quantify the statistical uncertainty around the point estimate and to allow an assessment of the precision of the estimates. Confidence intervals were interpreted as the range of values within which the true population incidence rate is expected to lie with a 95% probability under the assumed model. For observations with small counts (≤5 events), confidence intervals were calculated using the exact Poisson method, which provides more reliable interval estimates when the number of events is low. All analyses were performed using Microsoft Excel (2019 version, Microsoft Corporation, Redmond, WA, USA).

## 3. Results

Between 2019 and 2024, a total of 669 pertussis cases were reported in Tuscany in PREMAL. The period incidence rate was 18.19 cases per 100,000 inhabitants (IC 95%: 16.81–19.56), with substantial differences between individual years.

**Figure 1:**
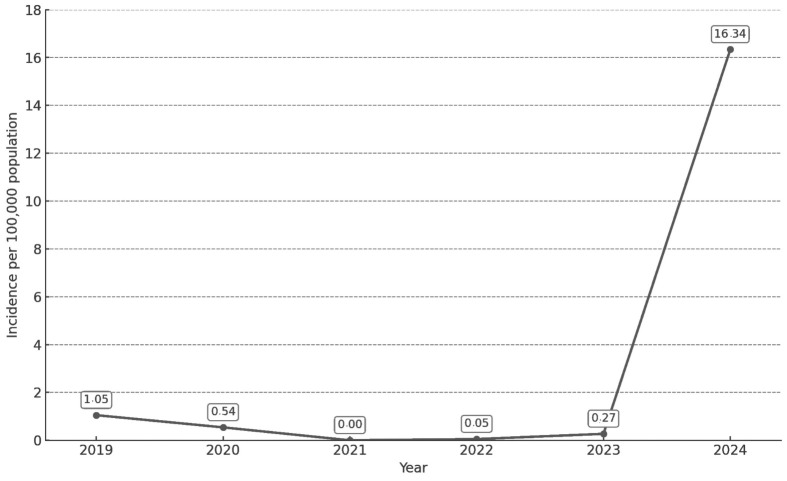
Distribution of annual pertussis incidence rates; Tuscany Region, 2019–2024.

In 2020, a decrease in notifications was observed, with an incidence rate of 0.54 cases per 100,000 inhabitants (IC 95%: 0.30–0.77) (20 cases), halving the number of cases reported in 2019 (39 cases, incidence: 1.05 per 100,000 (IC 95%: 0.72–1.37)). Between 2021 and 2023, incidence remained very low: 0.00 in 2021, 0.05 in 2022 (IC 95%: 0.01–0.18), and 0.27 per 100,000 inhabitants in 2023 (IC 95%: 0.10–0.44) (zero, two and 10 cases, respectively). The year 2024 was instead marked by a rapid growth to 598 notifications, accounting for 89% of all cases reported during the study period and reaching an incidence of 16.34 cases per 100,000 inhabitants (IC 95%: 15.03–17.65) (Figure 1).

No relevant differences were found in the case distribution between the two sexes. In 2024, incidence rates were 16.91 per 100,000 (IC 95%: 15.00–18.82) for males and 15.79 (IC 95%: 13.99–17.59) for females, with similar period rates in the entire 2019–2024 period (18.68 (IC 95%: 16.68–20.69) and 17.72 (IC 95%: 15.82–19.61) per 100,000, respectively).

When comparing local health units, the Tuscany Central area showed the highest incidence in 2024 (24.72 per 100,000 (IC 95%: 22.29–27.15)), followed by Tuscany North-West (12.61 (IC 95% 10.64–14.58)) and Tuscany South-East (5.44 (IC 95% 3.83–7.05)). This pattern was consistent across the period data, with values of 26.59 (IC 95%: 24.07–29.11), 15.40 (IC 95%: 13.22–17.57), and 5.87 (IC 95%: 4.21–7.53) per 100,000, respectively, for the entire examined period (Supplementary Table S1).

### 3.1. Age Distribution

Most pertussis cases in Tuscany during the 2019–2024 period were reported among individuals under 15 years of age (87.59%). The highest number of cases was recorded in the 10–14-year age group (361 cases; 53.96%), followed by children under the age of 5 years (114; 17.04%) and aged 5–9 years (111; 16.59%). Table 1 summarizes the total number of cases, the period incidence rate and the mean annual incidence rate for the most important age groups.

In terms of period incidence rate, the 10–14 group ranks first at 217.61 (IC 95%: 195.19–240.04) cases per 100,000; in second place we find the 0–4 age group (92.66 per 100,000 (IC 95%: 75.66–109.67)). Slightly lower rates were reported in the 5–9-year age group (74.82 per 100,000 (IC 95%: 60.91–88.74)), while individuals aged ≥15 years had the lowest incidence (2.56 per 100,000 (IC 95%: 2.01–3.11)).

**Figure 2:**
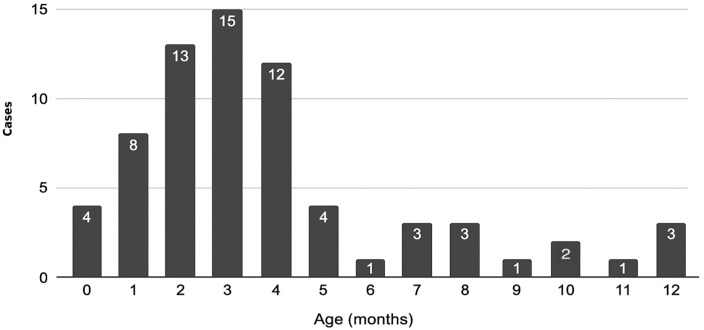
Distribution of pertussis cases by month of age among infants aged 0–12 months; Tuscany Region, 2019–2024.

Nevertheless, the examination of smaller age groups shows that infants under the age of 1 year were consistently the most affected group across the entire observation period, with a period incidence rate of 296.90 per 100,000 (IC 95%: 225.91–367.88), representing 10.01% of all cases alone. A more detailed analysis of infants (0–1 year) indicates that about 60% of cases occurred within the first 4 months of life (Figure 2).

The population over the age of 15 years accounted for only 12.41% of cases, with just a few cases per each 5-year age group. The most impacted categories include the 15–19 and 45–49 age groups, with 21 and 13 total cases respectively, mostly during the 2024 peak, reaching an incidence of 12.58 (IC 95% 7.79–19.23) and 4.46 (IC 95% 2.37–7.63) cases per 100,000 inhabitants respectively. Older adults showed an even lower number of infections, with only 16 cases reported among individuals over 65 years of age.

### 3.2. Time Trend of Age Distribution

**Figure 3:**
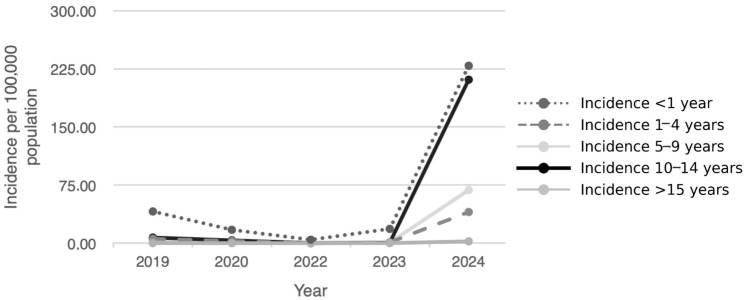
Distribution of age-specific pertussis incidence rates per 100,000 population, Tuscany Region, 2019–2024.

All age groups show a similar temporal trend, characterized by a progressive decline from 2019 to 2022, a slight increase in 2023 and a marked growth in 2024 (Figure 3). However, this increment was more pronounced in some groups compared to others.

Infants (<1 year) continue to be the category with the highest incidence across the entire period, but compared to the other pediatric age groups, they registered the smallest growth in 2024: a 176% increase against a 1751% increase in children aged 10–14 and 544% growth in the 5–9 age group. Similarly, incidence among adolescents aged ≥15 years and adults experienced a notable rise (964%), growing from 0.22 cases per 100,000 (IC 95%: 0.09–0.45) in 2019–2023 to 2.34 (IC 95%: 1.81–2.87) in 2024. Table 2 illustrates the change in incidence from 2019–2023 to 2024 for different age groups.

### 3.3. Geographical Distribution

**Figure 4:**
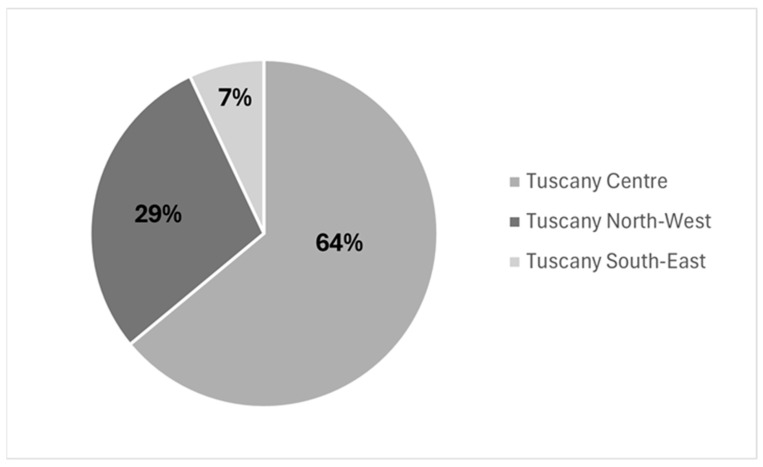
Geographical distribution of pertussis cases by Local Health Authority; Tuscany Region, 2019–2024.

The Central Tuscany Local Health Authority reported the majority of cases (428; 64% of the total 669 pertussis cases) with an incidence rate of 26.59 per 100.000 (IC 95%: 24.07–29.11) inhabitants, followed by the North-West Tuscany LHA (193; 29%) with 15.40 cases per 100.000 (IC 95%: 13.22–17.57) inhabitants and the South East Tuscany LHA (48; 7%) with 5.87 cases per 100.000 (IC 95%: 4.21–7.53) inhabitants (Figure 4), (Supplementary Table S1).

### 3.4. Hospitalizations

Overall, 84 cases required hospitalization (12.56% of the total). High percentages, but low absolute numbers, were observed in 2019 (25.6%; 10 cases; 0.27 cases per 100.000 inhabitants (IC 95%: 0.10–0.44)) and 2023 (60%; six cases; 0.16 cases per 100.000 inhabitants (IC 95%: 0.03–0.29)). In 2024, despite 598 notifications, only 10.4% of cases required hospitalization, with an incidence rate of 1.69 per 100,000 (IC 95%: 1.27–2.11) The hospitalization rate in the general population was therefore 2.3 per 100,000 inhabitants (IC 95%: 1.81–2.79) per year (Supplementary Table S2).

## 4. Discussion

After years of low incidence, Tuscany experienced a sudden increase in reported pertussis cases in 2024, similarly to what has been observed in other European and non-European countries. Pertussis is characterized by the periodic occurrence of epidemic peaks, but the 2024 outbreak displayed several features that diverge from previously recorded trends [9]. Firstly, the increase in reported cases was particularly pronounced in the last observed year, both in absolute terms and in relation to previous years: 598 cases in 2024 versus 32 total cases from 2020 to 2023. Even the last peak of the decade, recorded in 2017, accounted for only about one-fifth of the 2024 cases [11]. According to the available data, the age of the most affected groups increased, while no gender-based differences were detected. Moreover, despite the high number of cases, the proportion of hospitalizations in 2024 was markedly lower than that in previous years. These elements may indicate possible changes in the epidemiology of pertussis in Tuscany, similar to trends observed in other European countries; however, although the surveillance system can detect this situation, the lack of complete data hinders a clear interpretation of the epidemiological context.

Immunity to B. pertussis, whether natural or vaccine-induced, tends to wane over time (4–10 years), leading to the gradual formation of vulnerable groups, even in the context of high overall vaccination coverage [2,16]. This mechanism is considered one of the possible drivers of epidemic peaks [2,17,18]. Additionally, the latest ECDC report highlights the COVID-19 pandemic and a possible decline in vaccination coverage as contributing factors to the 2023–2024 epidemic peak. Several studies have investigated the impact of the pandemic on pertussis epidemiology [19]. As observed for other respiratory infections, the use of face masks, lockdowns, and social distancing may have temporarily reduced B. pertussis circulation, explaining the extremely low numbers during 2020–2022 [20,21]. Additionally, interruptions or delays in immunization programs during the pandemic may have contributed to the accumulation of susceptible individuals, who subsequently acquired the infection once preventive measures were relaxed.

Nevertheless, the rising number of reported cases in Tuscany could also be attributed to increased diagnostic sensitivity. After a decade of absence, in 2023, the ECDC weekly Communicable Disease Threats Report reintroduced the reporting of pertussis outbreaks in European countries, while in May 2024, a dedicated rapid risk assessment was published [22,23]. The renewed international and national awareness might have heightened attention to the disease.

The changes in the recorded hospitalization rate seem to point in this direction. Despite the rise in reported cases in 2024, only 10.4% required hospitalization, compared to 60% in 2023, 25% in 2021, and 30% in 2020. This decline may also indicate lower clinical severity of cases or more effective outpatient management. There are no further elements suggesting changes in disease severity, nor did Tuscany introduce significant management innovations that could justify a more efficient outpatient management strategy. In this context, it might be more plausible that a higher proportion of milder cases, which might previously have gone underreported, were detected. During the COVID-19 pandemic, non-severe cases may have been more difficult to recognize; conversely, the end of the emergency phase and heightened attention to respiratory infections may have generated the opposite effect. However, the high proportion of notifications with missing information on disease severity limited the ability to conduct an in-depth analysis of clinical severity and the associated hospital burden. Although this variable has been included in the surveillance system since 2024, the extent of missing data prevents a comprehensive assessment.

Geographical differences in reporting also deserve attention. Sixty-four percent of cases were reported in the Central Tuscany LHA, possibly reflecting higher transmission linked to greater population density (318.6 inhabitants/km^2^; >1.6 million) compared with the North-West (194 inhabitants/km^2^; 1.2 million) and South-East (70 inhabitants/km^2^; 0.8 million) LHAs [24] and also potentially indicating stronger reporting capacity in this area.

Another distinctive feature of the 2024 outbreak was the apparent epidemiological shift from children under 4 years to older children and adolescents. The highest incidence was recorded in the 10–14 age group, surpassing the traditionally more affected 0–4 age group (211 (IC 95%: 188.67–233.35) vs. 75.2 (IC 95%: 59.22–91.18) per 100,000). This phenomenon has been observed in other European countries such as Spain, France, and Denmark and has been attributed to the decline in pertussis immunity over time, followed by the progressive accumulation of susceptible individuals among the population during periods of low natural circulation of the pathogen [2,16,17,25,26]. Logistical challenges, organizational changes, and reluctance to attend healthcare facilities during the pandemic may have further reduced vaccine uptake, a phenomenon also observed in other countries, thereby increasing the pool of susceptible individuals [27].

However, two contextual factors should be considered: variable vaccination coverage and potential underreporting. The PREMAL surveillance system does not capture vaccination status; therefore, incidence estimates were calculated using the entire population as the denominator, regardless of immunity status. In Italy, pertussis vaccination has been mandatory for school attendance since 2017, with a schedule at 2, 4, and 10 months and a booster at 5 years. Due to waning immunity, additional boosters are recommended—though not mandatory—in adolescence (from 12 years) and every 10 years in adulthood. While infant vaccination coverage is high, uptake among adolescents and adults remains suboptimal (<95%) [28]. An Italian study estimated adult booster coverage at 10.6% in 2019, with substantial regional variation. In Tuscany, coverage among adolescents (16-year-old cohort) was 75.8% in 2023, increasing slightly to 79.5% in 2024 [29,30], possibly reflecting heightened awareness and vaccination efforts following the rise in reported cases.

Moreover, an analysis of hospitalized pertussis cases in Tuscany in 2024 found that 86.9% of affected adolescents between 12 and 16 years of age were either unvaccinated or had not received the adolescence booster. Vaccination delays were detected across all pediatric age groups, peaking in adolescence, with a median delay of 395 days between the first eligible vaccination date and the onset of the infection [31].

However, the lack of age-specific vaccination coverage data precludes direct comparisons and limits the ability to determine whether the higher incidence observed in older children reflects a true epidemiological shift. Interpretation is further constrained by likely substantial underreporting, particularly among adolescents and adults: only 62 cases in individuals > 20 years were reported between 2019 and 2024 (55 in 2024), including just 16 among those >65 years. Seroprevalence studies suggest that underreporting may reach 141-fold in adolescents and 3452-fold in adults [2]. Waning immunity and milder clinical presentations likely contribute to missed diagnoses, while improved detection of pauci-symptomatic infections may partly explain the increase in reported cases in the 10–14 age group [9].

Regardless of the quality and completeness of the surveillance information, vaccination coverage among adolescents and adults is known to be suboptimal [29,30]. Recent studies have investigated the role of adolescents and adults in pathogen transmission, and it is already well established that individuals in close contact with infants (family members and caregivers) represent their main source of infection [32,33,34].

According to the available data, in 2024, infants accounted for 10% of cases and represented the age group with the second-highest incidence. Sixty percent of affected infants were under 4 months of age, having acquired the infection before starting or completing the primary vaccination cycle. Infants are the most vulnerable group, not only to infection but also to complications and severe forms of the disease. The only available protective strategies are maternal vaccination during pregnancy and the cocoon strategy.

In Tuscany, pertussis vaccination is offered free of charge to all pregnant women between the 27th and 36th weeks of gestation, regardless of vaccination history or previous pregnancies. Maternal vaccination protects the newborn until the initiation of the primary vaccination series [35]. However, uptake remains low and uneven, generally below 50% [36]. None of the mothers of the 77 infants hospitalized in Tuscany in 2024 had received pertussis vaccination during pregnancy [31].

Cocooning, a strategy that aims to vaccinate all individuals in close contact with newborns, has so far also proven insufficient. Although family members (parents, siblings, and grandparents) and individuals involved in childcare have been identified as the most frequent sources of *Bordetella pertussis* infection, the cocooning approach remains inadequately implemented [37].

This study has several limitations. First, the PREMAL surveillance system does not include vaccination status, preventing the assessment of vaccine effectiveness or the role of incomplete immunization. Second, age-specific vaccination coverage data were not available for all groups, limiting comparisons between incidence and immunity levels. Third, substantial underreporting—particularly among adolescents and adults—may have affected the observed age distribution of the cases.

These findings highlight the need to strengthen pertussis surveillance, improve booster vaccination uptake among adolescents and adults, and increase maternal vaccination coverage to better protect infants, who remain the most vulnerable group to severe disease.

First, strengthening the surveillance system and improving data completeness are essential to better characterize pertussis epidemiology. The high proportion of notifications with missing information on clinical severity limited the assessment of disease severity and related hospital burden. Although introduced into the surveillance system in 2024, the current level of missing data precludes a comprehensive evaluation.

Similarly, the lack of data on vaccination status of reported cases and on vaccination coverage across all age groups prevented the evaluation of vaccine effectiveness and population susceptibility. Addressing these information gaps would improve the interpretation of surveillance data and support more targeted public health interventions.

Second, increasing uptake of adolescent booster doses and adult revaccination should represent a public health priority. Booster vaccination should be promoted without delay and integrated with strategies aimed at protecting newborns, such as maternal immunization during pregnancy and the cocooning strategy. In this context, collaboration with primary care providers—including general practitioners, pediatricians, obstetricians and gynecologists—is crucial both for improving case detection, particularly of mild or atypical infections, and for promoting vaccination uptake [38,39].

These findings suggest that the recent increase in pertussis cases may reflect both changes in transmission dynamics and improved case detection, highlighting the need for strengthened surveillance and sustained vaccination strategies across all age groups.

## 5. Conclusions

The 2024 pertussis outbreak in Tuscany was characterized by a sharp increase in reported cases compared with previous years, a shift in age distribution toward older children and adolescents, and a marked reduction in hospitalization rates—patterns also observed in several European countries and potentially explained by waning immunity, accumulation of susceptible individuals, pandemic-related disruptions, and increased diagnostic awareness. However, limitations in data completeness, lack of vaccination information, incomplete coverage data, and likely underreporting constrain interpretation, underscoring the need to strengthen surveillance and case detection, in addition to the implementation of targeted public health interventions aimed at improving booster vaccination uptake among adolescents and adults and increasing maternal vaccination coverage to reduce population susceptibility and better protect infants.

## Figures and Tables

**Table 1:** Distribution of pertussis cases, period incidence rate by age group and mean annual incidence rate; Tuscany Region, 2019–2024.

Age Groups	Number of Cases in 2019–2024 (% of the Total 669 Pertussis Cases)	Period Incidence Rate in 2019–2024 per 100,000	CI (95%)	Mean Annual Incidence Rate in 2019–2024	CI (95%)
**0–4 years**	114 (17.04%)	92.66	75.66–109.67	15.44	8.50–22.39
**<1 year**	67 (10.01%)	296.90	225.91–367.88	49.48	20.47–78.50
**1–4 years**	47 (7.03%)	46.79	33.41–60.16	7.80	2.34–13.26
**5–9 years**	111 (16.59%)	74.82	60.91–88.74	12.47	6.79–18.15
**10–14 years**	361 (53.96%)	217.61	195.19–240.04	36.27	27.11–45.43
**>15 years**	83 (12.41%)	2.56	2.01–3.11	0.43	0.20–0.65
**15–19 years**	21 (3.14%)	12.58	7.79–19.23	2.10	1.56–3.85
**20–24 years**	1 (0.15%)	0.60	0.02–2.33	0.10	0.00–0.67
**25–29 years**	3 (0.45%)	1.73	0.36–5.06	0.29	0.07–1.01
**30–34 years**	6 (0.90%)	3.17	1.16–6.91	0.53	0.23–1.38
**35–39 years**	7 (1.05%)	3.39	1.36–6.91	0.56	0.27–1.40
**40–44 years**	5 (0.75%)	2.05	0.66–4.78	0.34	0.13–0.96
**45–49 years**	13 (1.94%)	4.46	2.37–7.63	0.74	0.47–1.53
**50–54 years**	3 (0.45%)	0.99	0.20–2.88	0.16	0.04–0.58
**55–59 years**	4 (0.60%)	1.37	0.37–3.51	0.23	0.07–0.70
**60–64 years**	4 (0.60%)	1.59	0.43–4.06	0.26	0.09–0.81
**>65 years**	16 (2.39%)	1.68	0.96–2.72	0.28	0.19–0.54

**Table 2:** Distribution of period (2019–2023), 2024 pertussis incidence per 100,000 and mean annual incidence rate (2019–2023) and relative percentage increase by age group in the Tuscany Region.

Age	Period Incidence Rate 2019–2023	CI (95%)	Incidence 2024	CI (95%)	Percentage Increase: Period Incidence Rate in 2019–23 to Incidence in 2024	Mean Annual Incidence Rate in 2019–2023	CI (95%)	Percentage Increase: Mean Annual in 2019–23 to Incidence in 2024
**<1 year**	83.02	49.98–129.64	228.94	164.25–293.64	+176%	16.60	10.00–25.93	1279%
**1–4 years**	9.79	4.70–18.01	40.17	27.23–53.11	+310%	1.96	0.94–3.60	1949%
**5–9 years**	10.65	6.08–17.29	68.54	54.76–82.32	+544%	2.13	1.22–3.46	3118%
**10–14 years**	11.40	6.86–17.80	211.01	188.67–233.35	+1751%	2.28	1.37–3.56	9155%
**>15 years**	0.22	0.09–0.45	2.34	1.81–2.87	+964%	0.04	0.02–0.09	5750%

## Data Availability

Data from the national surveillance system (PREMAL).

## Supplementary Materials

The following supporting information can be downloaded at: https://www.mdpi.com/article/10.3390/pathogens15030326/s1, Table S1: Number of Pertussis Cases and Incidence Rates per 100,000 in Tuscany (Italy), 2019–2024; Table S2: Hospitalizations due to Pertussis: Number of Cases and Incidence Rates in Tuscany (Italy), 2019–2024.

## Abbreviations

The following abbreviations are used in this manuscript:

PREMAL	Sistema per la sorveglianza delle malattie infettive (national surveillance system).
LHA	Local Health Authority.
ISTAT	Istituto Nazionale di Statistica (National Institute of Statistics).
ISS	Istituto Superiore di Sanità (National Health Institute).
ECDC	European Centre for Disease Prevention and Control.
