# Supplementary material for: The Resurgence of Pertussis in Tuscany (Italy): A Six-Year Retrospective Epidemiological Analysis
Source: Pathogens. 2026 Mar 18;15(3):326. doi: 10.3390/pathogens15030326 (PMC13029293; doi:10.3390/pathogens15030326)
Supplement: Supplementary file 1 [file pathogens-15-00326-s001.zip › pathogens-4180969-supplementary.pdf]

Supplementary Materials

Supplementary Table S1: Number of Pertussis Cases and Incidence Rates per 100,000 in Tuscany (Italy), 2019–2024

Population		2019	2020	2022	2023	2024	Period (19-24)	Mean annual incidence rate (19-24)	Period (19-23)	Mean annual incidence rate (19-23)
General population (M+F)	N. of cases	39	20	2	10	598	669		71	
	Incidence	1.05	0.54	0.05	0.27	16.34	18.19	3.03	1.93	0.39
		(0.72 - 1.37)	(0.30-0.77)	(0.01-0.18)	(0.10-0.44)	(15.03-17.65)	(16.81-19.56)	(2.47-3.59)	(1.48-2.38)	(0.19-0.59)
Male	N. of cases	16	10	2	4	301	333		32	
	Incidence	0.89	0.56	0.11	0.23	16.91	18.68	3.11	1.79	0.36
		(0.45-1.33)	(0.21-0.91)	(0.01-0.40)	(0.06-0.59)	(15.00-18.82)	(16.68-20.69)	(2.29-3.93)	(1.17-2.42)	(0.08-0.64)
Female	N. of cases	23	10	0	6	297	336		39	
	Incidence	1.20	0.52	0	0.32	15.79	17.72	2.95	2.05	0.41
		(0.71-1.69)	(0.20-0.84)	0	(0.06-0.58)	(13.99-17.59)	(15.82-19.61)	(2.18-3.73)	(1.41-2.70)	(0.12-0.70)
LHA Central Tuscany	N.of cases	19	7	1	4	397	428		31	
	Incidence	1.18	0.44	0.06	0.25	24.72	26.59	4.43	1.93	0.39
		(0.65-1.71)	(0.12-0.76)	(0.00-0.33)	(0.07-0.64)	(22.29-27.15)	(24.07-29.11)	(3.40-5.46)	(1.25-2.60)	(0.08-0.69)
LHA North-West	N. of cases	17	13	1	5	157	193		36	
	Incidence	1.34	1.03	0.08	0.40	12.61	15.40	2.57	2.87	0.57
		(0.70-1.98)	(0.47-1.59)	(0.00-0.45)	(0.13-0.93)	(10.64-14.58)	(13.22-17.57)	(1.68-3.45)	(1.93-3.81)	(0.15-0.99)
LHA South-East	N. of cases	3	0	0	1	44	48		4	
	Incidence	0.36	0	0	0.12	5.44	5.87	0.98	0.49	0.10
		(0.07-1.05)	0	0	(-0.00-0.67)	(3.83-7.05)	(4.21-7.53)	(0.30-1.66)	(0.13-1.25)	(0.03-0.25)
< 1 year	N. of cases	10	4	1	4	48	67		19	

	Incidence	40.94	17.23	4.41	18.43	228.94	296.90	49.48	83.02	16.60
	CI (95%)	(15.57-66.31)	(4.69-44.11)	(0.11-24.59)	(5.02-47.18)	(164.25–293.64)	(225.91 - 367.88)	(20.47 - 78.50)	(49.98–129.64)	(10.00–25.93)
**0-4 years**	N. of cases	16	7	1	5	85	114		29	
	Incidence	12	5.4	0.8	4.3	75.2	92.66	15.44	23.20	4.64
	CI (95%)	(6.13-17.87)	1.38-9.42)	(0.02 - 4.62)	(1.39 - 10.00)	(59.22-91.18)	(75.66 - 109.67)	(8.50 - 22.39)	(14.76-31.64)	(0.86-8.42)
**5-9 years**	N. of cases	8	6	1	1	95	111		16	
	Incidence	5.08	3.89	0.68	0.70	68.54	74.82	12.47	10.65	2.13
	CI (95%)	(1.56-8.60)	(0.78-7.00)	(0.02 - 3.80)	(0.02 - 3.92)	(54.76–82.32)	(60.91 - 88.74)	(6.79 - 18.15)	(6.08–17.29)	(1.22–3.46)
**10-14 years**	N. of cases	12	6	0	1	342	361		19	
	Incidence	7.22	3.60	0	0.60	211.00	217.61	36.27	11.40	2.28
	CI (95%)	(3.14-11.30)	(0.72-6.48)	0	(0.02 - 3.37)	(188.67–233.35)	(195.19 - 240.04)	(27.11 - 45.43)	(6.86–17.80)	(1.37-3.56)
**> 15 years**	N. of cases	3	1	0	3	76	83		7	
	Incidence	0.09	0.03	0	0.09	2.34	2.56	0.43	0.22	0.04
	CI (95%)	(0.02 - 0.27)	(0.00 - 0.17)	(0.00 - 0.17)	(0.02 - 0.27)	(1.81–2.87)	(2.01 - 3.11)	(0.20 - 0.65)	(0.09–0.45)	(0.02–0.09)

Supplementary Table S2: Hospitalizations due to Pertussis: Number of Cases and Incidence Rates in Tuscany (Italy), 2019–2024

**Hospitalization**		**2019**	**2020**	**2022**	**2023**	**2024**	**Period (19-24)**	**Mean annual incidence rate (19-24)**
**Total**	N. of hospitalization cases	10	6	0	6	62	84	
	% hospitalization/ total of cases of the year	25.6%	30.0%	0.0%	60.0%	10.4%	12.56%	
	Incidence per 100,000	0.27	0.16	0	0.16	1.69	2.3	0.38
	IC 95%	0.10 – 0.44	0.03 – 0.29	0	0.03 – 0.29	1.27 – 2.11	1.81 – 2.79	0.30 – 0.46
